# From security to solidarity: The normative foundation of a global pandemic treaty

**DOI:** 10.7189/jogh.12.03025

**Published:** 2022-05-14

**Authors:** Po-Han Lee, Ming-Jui Yeh

**Affiliations:** National Taiwan University, Institute of Health Policy and Management, Taipei, Taiwan

“We are all in this together”. Political leaders, research communities, and civil society organisations have often made this statement during the COVID-pandemic in their calls for global and social solidarity. The statement has two layers of connotation. Empirically, a scenario exists in which we are facing a common threat, despite the unequal distribution of burden and consequence. Normatively, that scenario requires us to recognise our interconnectedness and do something together about the threat. Any type of joint action, based on our interdependent and complex relationships in the scenario, will rely on our aggregate capacity as a collective [1].

The idea of solidarity, mentioned repeatedly in the early stage of the COVID-19 pandemic caused by severe acute respiratory syndrome coronavirus 2 (SARS-CoV-2), has somehow been missing from policy discussion until now [2]. Bringing attention back to solidarity is key in renovating the global pandemic response strategy. The World Health Organisation (WHO) member states just adopted resolution SSA2(5) on “The World Together” on December 1, 2021, which establishes an intergovernmental body for drafting and negotiating new legal instruments for pandemic prevention, preparedness, and response.

In this essay, we hope to offer a stronger justification to echo the World Health Assembly (WHA) resolution that stresses the principle of solidarity with all peoples and countries. To do so, we first identify the discursive shifts in global health cooperation and argue that global solidarity is necessary for the promotion of global health and the realisation of global health law in the post-COVID era. Such a sense of global solidarity is not only ethically necessary for ensuring commitment in carrying costs to assist other equal members [3], but also practically necessary, in order to promote and incentivise the seeking of international support and cooperation.

## UNANTICIPATED EVENTS FOREGROUNDING MOMENTS FOR TRANSFORMATION

The use of international legal instruments in controlling emerging infectious diseases and detecting outbreaks has a long history and has always been the core task of international health law. It could be argued that, considering the quarantine measures practised and acquiesced by all European cities against the “Black Death” in the 14th century, an inter-polity pandemic response regime has existed longer than the conception of state sovereignty and the Westphalian international system.

**Figure:**
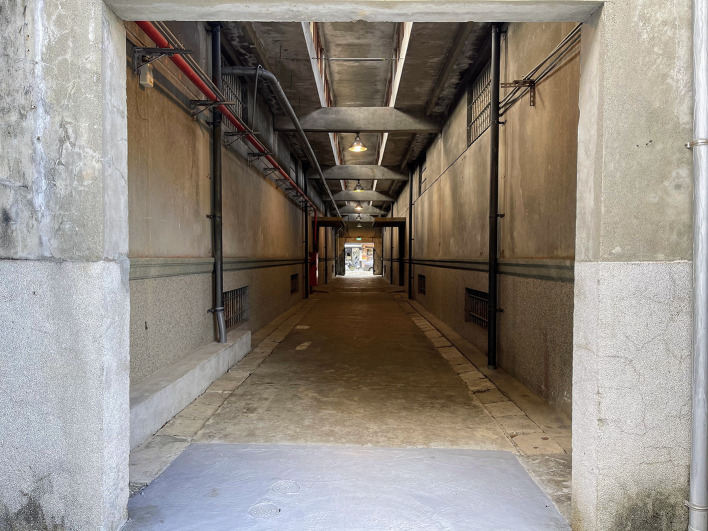
Photo: The empty Hualien Tourism Sugar Factory during the pandemic time. Although there is light at the end of the channel, what is out there remains uncertain. Source: From Po-Han Lee’s collection, used with permission).

We are standing now at the conjunction in which painful experiences of COVID-19 have pressed us to reconsider the normative foundation and efficiency of the old approach to designing global pandemic strategy [4]. Societies feel less distant from one another during the pandemic, so the affirmation of solidarity is similar to the calls for global health governance by involving non-state actors and stakeholders and holding them accountable beyond state-centrism in the aftermath of the Cold War.

Despite the several calls for updating the International Health Regulations (IHR) in the 1990s, they were not taken seriously enough until the world witnessed the 2001 anthrax attacks and the emergence of bioterrorism and the 2003 SARS (severe acute respiratory syndrome) outbreak. Against this background, the current IHR were finally revised in 2005 by the 58th WHA.

## INEFFECTIVENESS OF IHR 2005 UNDERPINNED BY THE SECURITY DISCOURSE

The 2005 IHR were innovative at their time, taking an “all-risks approach” that abstracts public health threats beyond specific diseases. The instrument conceptualises public health emergencies of international concern (PHEIC) underpinned by the idea of global health security. It requires the states – the main actors in disease outbreak response – to develop their own core public health capacities and authorises the WHO Director-General to receive unofficial information and determine with an Emergency Committee whether a PHEIC is found, and issue non-binding recommendations.

The COVID-19 outbreak is the 6th PHEIC, which was declared on January 31, 2020, based on the IHR 2005, a month after the first case was reported by China to the WHO. Analyses have identified the weaknesses of the legal instrument prior to and during the COVID-19 pandemic, such as the lack of consistency and transparency in the rationales of evaluating and determining whether an outbreak constitutes a PHEIC, despite the decision instrument contained in Annex 2 of the 2005 IHR [5,6].

Based on the security discourse, the 2005 IHR have been created to prevent and control the international spread of disease. Under the Westphalian framework, a nation-state is presumed to have the exclusive capacity to contain and respond to emerging public health emergencies [7]. A state’s sovereign right to make and implement relevant laws and rules is also enshrined in the 2005 IHR. In the face of the COVID-19 pandemic, the state-centric security discourse did not work out or has been insufficient in promoting global awareness of the imminent threat posed by the novel disease.

### An emerging momentum for rethinking global health governance

In May and November 2021, the Independent Panel for Pandemic Preparedness and Response, established by the WHO Director-General, mapped out a critical chronology regarding how and why the COVID-19 outbreak became a pandemic and explored various action areas for improving the existing system. Similar to many other proposals, the Independent Panel recommended incorporating the One Health perspective in the redesigned system against the risk posed by the Anthropocene and adopting a highly precautionary approach to respiratory illnesses.

With this approach, respiratory disease outbreaks should always be taken as potential PHEICs unless strong evidence suggests otherwise, considering the global health consequences of not declaring a PHEIC. To realise sufficient precaution, verification mechanisms and whistle-blower protection are necessary. It is also important to re-incentivise the states or communities affected by disease outbreaks to regard cooperation as practising good global citizenship rather than admitting to having poor capacities.

Particularly in this regard, the 2005 IHR have primarily focused on public health capacity building in developing countries that has given a false sense of security to other parts of the world. This resulted in delayed national responses from societies that were overconfident in the strength of their health systems and who were yet underprepared for pandemics like COVID-19. Capacity does not guarantee safety, but rather induces complacency [8]. Therefore, we consider that the discourse of global solidarity is required for forging an ethically preferable global pandemic response regime, whether in terms of revising the IHR or creating a new treaty.

## CONCLUSION

At the critical moment where the transformation of global health governance is about to happen, we consider that both the sense of feeling prepared prior to an outbreak and the sense of urgency when it occurs derive from the fact that we are and will still be in this together. These two senses are what we understand as global solidarity for emerging disease outbreaks, without which the institutional redesign may still fail. The belief in global solidarity, shared between global citizens and the nation-states they constitute domestically, includes the dimensions of self-interest and the global public good. The former comes from the expectation for the boomerang effect of sharing, while the latter accumulates primary and side benefits from sharing burden and effort.

To complement, if not replace, the security and development discourses, the solidarity discourse based on the recognition of interconnectedness and interdependence is necessary for improving global governance. Ethically, such a sense of connection would facilitate the willingness to redistribute responsibility for just global health, which entails accountability and transparency following the acknowledgement of common threats and pursuing collective actions [9]. Practically, feeling solidarity is also crucial to changing the perspective on international cooperation from the “not until” mentality (submission to *international* governance) to an “as long as” attitude (exercise of *global* citizenship). This is what the world has needed and is now more urgent than ever.
